# Balancing act: optimizing blue light for melanogenesis while minimizing cellular damage in primary human skin cells

**DOI:** 10.3389/fphys.2024.1513054

**Published:** 2025-01-09

**Authors:** Augustin C. Barolet, Brice Magne, Karel Ferland, Natallia E. Uzunbajakava, Daniel Barolet, Lucie Germain

**Affiliations:** ^1^ Regenerative Medicine Division, CHU de Quebec – Université Laval Research Centre, Quebec City, QC, Canada; ^2^ Centre de recherche en organogénèse expérimentale de l’Université Laval (LOEX), Université Laval, Quebec City, QC, Canada; ^3^ RoseLab Skin Optics Research Laboratory, Laval, QC, Canada; ^4^ Department of Surgery, Faculty of Medicine, Université Laval, Quebec City, QC, Canada; ^5^ Department of Hybrid Printed Electronics, TNO at the Holst Centre, Eindhoven, Netherlands; ^6^ Dermatology Division, Department of Medicine, McGill University Health Centre, Montreal, QC, Canada

**Keywords:** blue light, cell viability, melanogenesis, melanocytes, alpha-MSH, keratinocytes, pigmentation, phototherapy

## Abstract

**Introduction:**

Recent findings show that visible light, particularly blue light, stimulates melanogenesis in human skin, though the underlying mechanisms remain debated. This study aimed to determine the cell damage threshold of non-ionizing blue light on keratinocytes while preserving their ability to stimulate melanogenesis.

**Methods:**

Human keratinocytes (N = 3) and melanocytes (N = 3) were isolated from skin samples of varying Fitzpatrick skin phototypes and irradiated with blue light (λpeak = 457 nm) and UVA light (λpeak = 385 nm). Cellular metabolic activity was assessed using the AlamarBlue HS assay, α-Melanocyte-Stimulating Hormone (α-MSH) production by keratinocytes was quantified using ELISA, and Western blotting was used to assess pro-melanogenic factor expression in melanocytes.

**Results:**

High blue light intensity (50 mW/cm^2^, 50 J/cm^2^) and UVA light (15 mW/cm^2^, 20 J/cm^2^) significantly reduced cellular metabolic activity, with a 0.86 ± 0.055 and 0.60 ± 0.031 (mean ± SD) fold decrease compared to their respective sham by day 7. In contrast, moderate blue light intensities (5–15 mW/cm^2^, 10–20 J/cm^2^) preserved cellular metabolic activity while stimulating α-MSH production, with an optimal balance achieved at 10 mW/cm^2^, 15 J/cm^2^ (1.14 ± 0.046 fold increase relative to sham on day 7). Co-culture experiments confirmed that irradiated keratinocytes enhanced melanogenesis in melanocytes via paracrine signaling, increasing the expression of Tyrosinase and Dopachrome Tautomerase (DCT). Direct blue light irradiation on melanocytes also increased pigmentation without significant cellular damage.

**Discussion:**

Moderate-intensity blue light at 10 mW/cm^2^, 15 J/cm^2^ effectively stimulates melanogenesis while maintaining cellular metabolic activity in both keratinocytes and melanocytes, offering a promising, safe approach for blue light therapies targeting pigmentation disorders.

## Highlights

• *Optimal Light Parameters for Safety and Efficacy*: The study identifies blue light parameters (10 mW/cm^2^, 15 J/cm^2^) that effectively stimulate melanogenesis while maintaining cell metabolic activity, providing crucial insights for therapeutic applications.
• *Biphasic Dose-Response in Melanogenesis*: Moderate blue light intensities (5 mW/cm^2^, 10 J/cm^2^ to 15 mW/cm^2^, 20 J/cm^2^) enhance melanogenesis without significant cytotoxicity, while the highest intensitiy (50 mW/cm^2^, 50 J/cm^2^) induce cytotoxicity, underscoring the importance of precise dosing.
• *Role of Cell Interactions in Melanogenesis*: Co-culture experiments demonstrated that irradiated keratinocytes enhanced melanogenesis in melanocytes via paracrine signaling, offering insights into the cellular mechanisms of melanogenesis under blue light exposure.
• *Foundation for Clinical Application*: These results lay the groundwork for developing blue light-based therapies for pigmentation disorders.

## Introduction

Recent evidence shows that visible light has the ability to stimulate melanogenesis in human skin (Moreiras et al., 2021a; Moreiras et al., 2021b). However, the exact mechanism underlying this phenomenon remains controversial. We know that keratinocytes regulate melanocyte function, notably through the canonical melanogenesis pathway via paracrine mechanisms (Hirobe, 2014). Other studies suspected a direct effect of light on melanocytes via their photolabile opsin receptors (OPNs), such as OPN1-SW, 3 and 5 (Moreiras et al., 2021b; Castellano-Pellicena et al., 2019; Ozdeslik et al., 2019; Regazzetti et al., 2018; Olinski et al., 2020). A combination of both effects is also possible, suggesting the need for further studies to elucidate this visible light-related stimulation in melanogenesis.

Shin et al. demonstrated that the effect of UVB on melanogenic stimulation was greater in a coculture of melanocytes and keratinocytes compared to a monoculture of melanocytes (Shin et al., 2012). In the absence of keratinocytes, the melanocytes were less stable, meaning they lacked the necessary signals to maintain their differentiation and function, resulting in insufficient melanin synthesis. The presence of keratinocytes provided critical paracrine factors that supported the stability and proper differentiation of melanocytes, allowing for effective melanin production.

Regazzetti et al. observed that primary cultures of melanocytes were greatly stimulated by blue light (Regazzetti et al., 2018). They investigated the role of opsin 3 receptors in blue light-induced stimulation and concluded that opsin 3 was primarily responsible for the modulation of differentiation and melanogenesis, with no involvement of keratinocytes. In contrast, Ozdeslik et al. demonstrated that opsin 3 activation inhibited melanocortin 1 receptor (MC1R) signaling in primary melanocyte cultures, leading to the conclusion that opsin 3 stimulation abrogated melanogenesis (Ozdeslik et al., 2019). These conflicting findings highlight the complexity of melanocyte responses to light, and further research may be needed to resolve these differences in interpretation.

Paracrine signaling between keratinocytes and melanocytes has long been recognized as essential for melanogenesis (Seiberg, 2001). A recent study suggests that small extracellular vesicles released by human epidermal keratinocytes are key regulators of melanocyte functions, such as melanosome maturation, dendrite formation, and pigment transfer, thus enhancing intercellular communication within the skin (Prospéri et al., 2024; Lo Cicero et al., 2015).

However, concerns have been raised about the risks associated with blue light exposure, particularly regarding cellular damage. *In vitro* studies using 2D human cell cultures have suggested that blue light can cause DNA damage, photodamage to fibrillin, elastin, and collagen, and induce apoptosis through altered protein activities, including matrix metalloproteinases (MMPs) (Schütz, 2021; Coats et al., 2021; Christensen et al., 2021; Dong et al., 2019). Specifically, increased MMP activity can degrade extracellular matrix components, leading to the disruption of cellular adhesion and structural integrity, which can trigger apoptotic pathways. Conversely, *in vivo* studies using intact human skin biopsies have not demonstrated these hallmarks of photodamage. Moreiras et al. (2021b) reported distinct light stimulation patterns of pigmentation in biopsies from patients with different Fitzpatrick skin phototypes (SPT). Researchers stimulated the pigmentation of these biopsies using UVR (λmax: 311 nm), blue (λpeak: 450 nm), and green (λpeak: 530 nm) light. All three wavelengths were able to increase pigmentation. Surprisingly, in fair-skinned subjects (SPT-I to SPT-III) blue and green light spectra stimulated skin pigmentation more effectively than UVR in fair skin biopsies (SPT-I to SPT-III). Additionally, no skin damage was observed when using these non-ionizing wavelengths compared to UVR. Discrepancies about whether blue light causes photodamage and how keratinocytes and melanocytes interact during melanogenesis highlight the limitations of previous studies. Hence, it remains crucial to determine the cell damage threshold of non-ionizing blue light, particularly on keratinocytes. To harness the therapeutic potential of blue light while minimizing risks, it is also essential to identify the optimal light parameters that can safely stimulate pigmentation in the skin without causing cellular damage (Uzunbajakava et al., 2022).

The study aimed to establish the cell damage threshold for non-ionizing blue light on keratinocytes, while ensuring their ability to stimulate melanogenesis. This was evaluated using the Alamar Blue HS assay to assess cell metabolic activity, and thereby indirectly, their cytotoxicity, as well as through α-Melanocyte-Stimulating Hormone (α-MSH) quantification using ELISA to evaluate keratinocyte response. The optimal light parameters ensuring both safety and efficacy were then applied to co-cultures of keratinocytes and melanocytes, with melanogenesis stimulation assessed through Western blotting for pro-melanogenic factors.

## Materials and methods

### Cell isolation and culture

Human cells were procured from healthy skin samples removed during surgical procedures. This included keratinocytes and melanocytes isolated from skin samples with SPT II and SPT IV skin types. Specimens were obtained from light-skinned (N = 3) and dark-skinned (N = 1) donors aged between 1 month and 69 years (see Table 1 for detailed donor information). The study protocol was approved on 12 May 2022, by the Research Ethics Committee for the Protection of Human Subjects (N2023-6411) at CHU de Québec-Université Laval. A signed consent form was obtained from each donor or their parents prior to their minor surgery for the collection of their skin samples, in accordance with the ethical principles outlined in the Declaration of Helsinki. To isolate keratinocytes and melanocytes, skin fragments were incubated in 500 μg/mL of thermolysin (Sigma-Aldrich, St-Louis, MO, United States) in HEPES buffer [10 mM of 4-(2-hydroxyethyl)-1-piperazine ethane sulfonic acid (MP Biomedicals Inc., CA, United States), 6.7 mM of potassium chloride, 142 mM of sodium chloride, and 1 mM of calcium chloride] at 4°C overnight. The epidermis was carefully detached from the dermis with the use of forceps. Keratinocytes and melanocytes were dissociated from the epidermis in a trypsin/EDTA solution (0.05% trypsin 1–500 [Gibco, Waltham, MA, United States] and 0.01% EDTA/disodium salt in phosphate-buffered saline) at 37°C for 25 min.

**TABLE 1:** Donor information of skin cell populations.

Id	Cell type	Source	Donor’s sex	Donor’s age	Fitzpatrick index
Population 1	Keratinocytes and Melanocytes	Foreskin	Male	<1 month	IV
Population 2	Keratinocytes	Facelift	Female	56 years	II
Population 3	Keratinocytes and Melanocytes	Breast Surgery	Female	69 years	II
Population 4	Melanocytes	Abdominoplasty	Female	46 years	II

The feeder layer fibroblasts were isolated from the dermis (foreskin) of a 10-day-old donor, which had been previously separated from the epidermis using a thermolysin solution, by incubating the tissue in a collagenase H solution (0.125 U/mL; Roche Diagnostics, Laval, Quebec, Canada) for 3 h at 37°C (Le-Bel et al., 2019). The dissociated fibroblasts were cultured in Dulbecco-Vogt modification of Eagle’s medium (DMEM) containing 10% fetal calf serum (HyClone, Wilmington, DE, United States) and antibiotics (penicillin at 100 U/mL and gentamicin at 0.025 mg/mL). Fibroblasts were expanded from passages 2 to 6, mitotically inactivated through gamma irradiation (6,000 rad), and frozen at −80°C in serum containing 10% dimethyl sulfoxide (DMSO, Sigma-Aldrich, St-Louis, MO, United States) as described (Rochon et al., 2009). Before use, irradiated fibroblasts were thawed and seeded at 8,000 cells/cm^2^ in T75 flasks using a medium consisting of a 3:1 mixture of DMEM and Ham’s F12 (Invitrogen, Waltham, MA, United States) supplemented with 5% Fetal Clone II serum (HyClone, Wilmington, DE, United States), 5 μg/mL insulin (Sigma-Aldrich, St. Louis, MO, United States), 0.4 μg/mL hydrocortisone (Calbiochem, San Diego, CA, United States), 1 × 10^−6^ M isoproterenol (Sigma-Aldrich, St. Louis, MO, United States), 10 ng/mL epidermal growth factor (EGF), and antibiotics referred to as keratinocyte medium. They were then cultured alone for at least 1 week without changing the cell medium to allow the accumulation of factors favorable for the keratinocyte proliferation. Feeder fibroblasts could be maintained in culture for up to 1 month, with the cell media refreshed weekly (Le-Bel et al., 2019; Bisson et al., 2013).

Keratinocytes were cultured as previously described (Cortez Ghio et al., 2019). Briefly, keratinocytes were seeded on the mitotically inactivated feeder fibroblasts in the keratinocyte medium. Cell media was changed every other day until the cells reached 80% confluence. Melanocytes were isolated from keratinocytes by culturing the epidermal cells for 2 days in melanocyte medium (Melanocyte Basal Medium [Lonza, Basel, Switzerland]) supplemented with 0.5% fetal bovine serum (HyClone, Wilmington, DE, United States), calcium, bovine pituitary extract, recombinant human basic fibroblast growth factor, recombinant human insulin, hydrocortisone, phorbol myristate acetate, penicillin, and gentamicin, along with 0.1 mg/mL geneticin (G418; Sigma Chemicals, St. Louis, MO, United States). All supplements were added at the concentrations determined and provided by Lonza, except for the geneticin (Goyer et al., 2019). After 2 days, geneticin was removed, and the melanocytes were cultured in melanocyte medium without geneticin.

### Description of LED module

**FIGURE 1:**
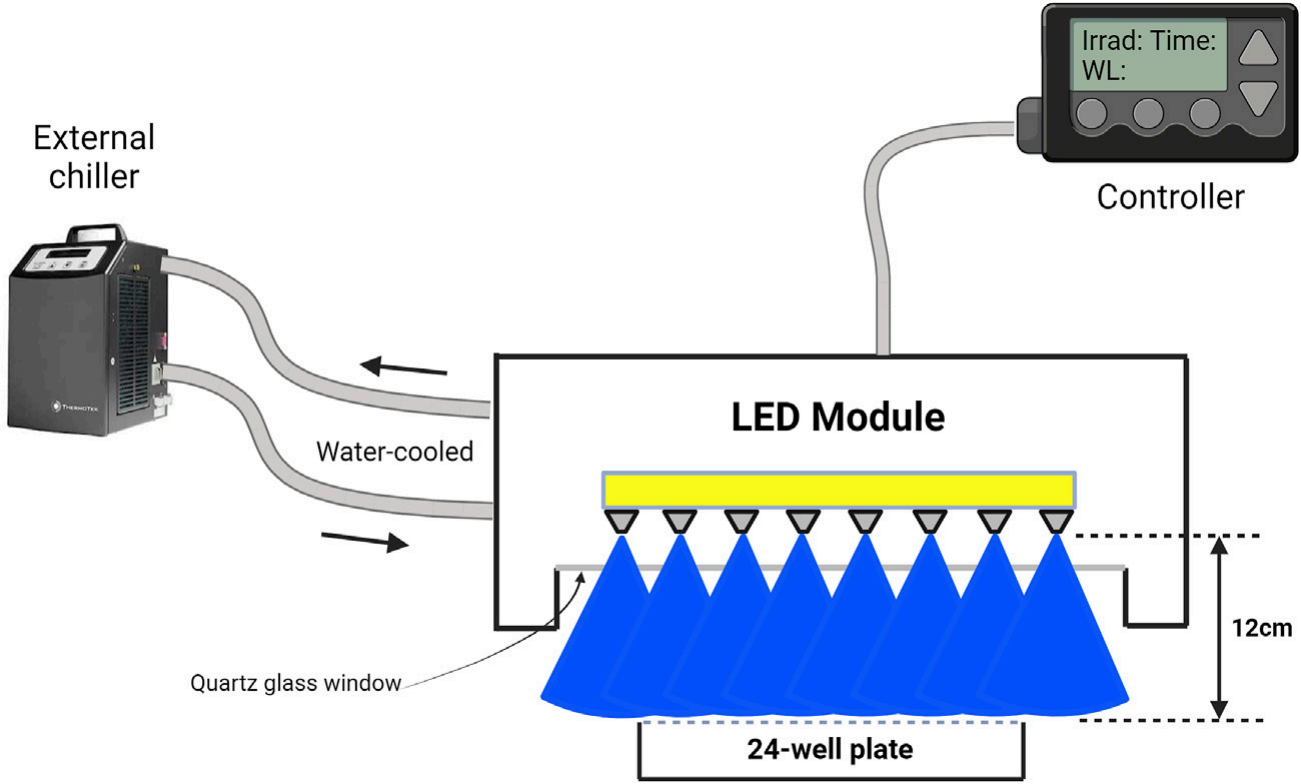
Schematic representation of an LED-based irradiation device designed for use within a cell culture flow hood. The water-cooled high-power light module comprises blue LEDs (λpeak = 457 nm ± 20 nm bandwidth) (shown here) and UVA LEDs (λpeak = 385 nm ± 10 nm bandwidth) arranged in rows of LEDs. The LEDs are positioned above the cell culture plates to ensure uniform irradiation. The distance between the LED array and the cell surface is precisely set at 12 cm, as confirmed by pre-calibration using ImageJ software to assess light homogeneity across the 24-well plate (shown here) or T75/T25 cell culture flasks. Exposure time and irradiance levels were controlled using a dedicated interface. All light treatments were conducted within the sterile environment of the cell culture flow hood to prevent contamination, and fresh media was added post-irradiation to maintain cell viability. This figure was illustrated with BioRender.

**FIGURE 2:**
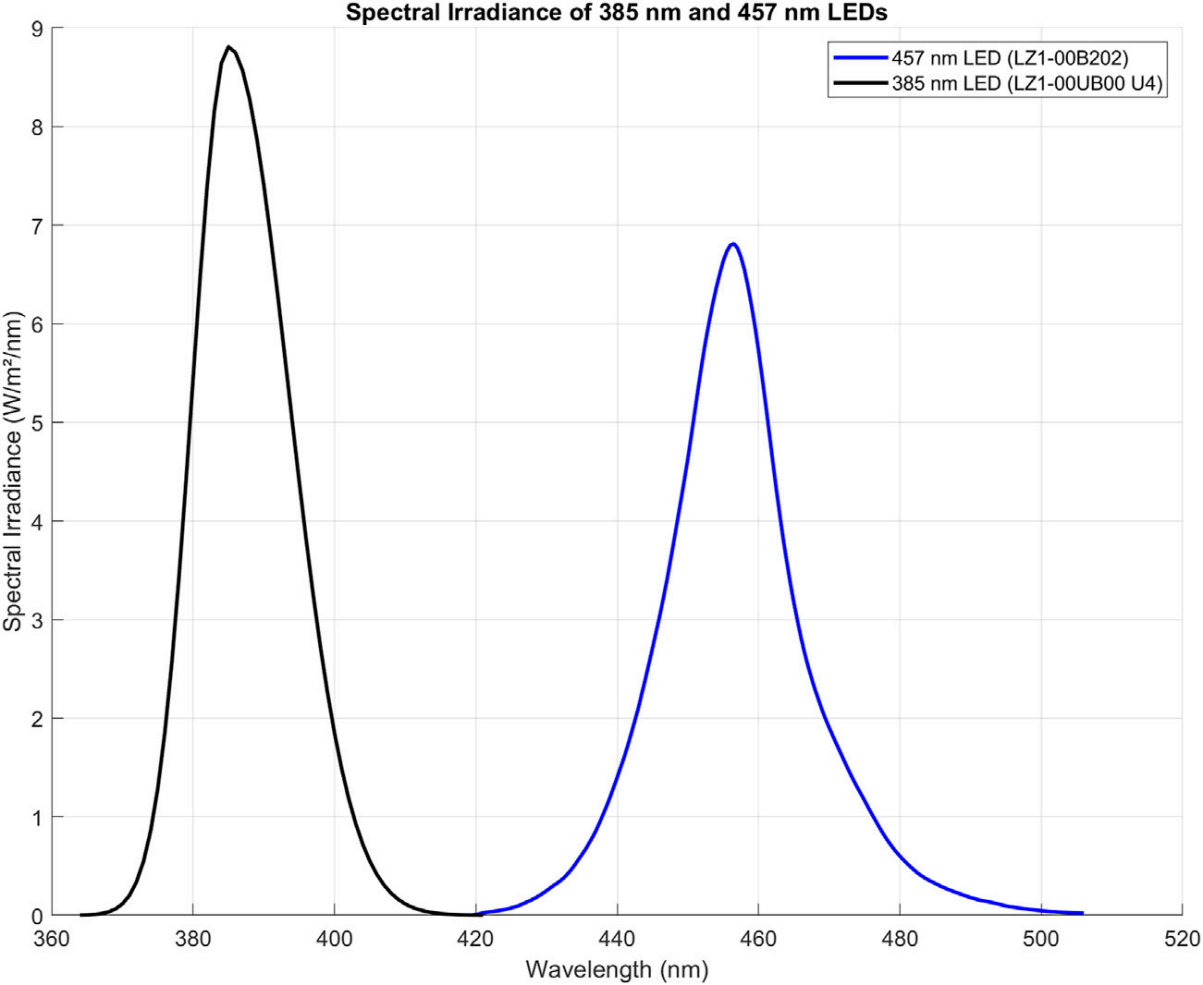
Spectral irradiance of 385 nm (LZ1-00UB0RU4) and 457 nm (LZ1-00B202) LEDs, measured at 12 cm from the spectrophotometer probe (OceanOptics, High-speed Spectrometers, SR2). Both LEDs were calibrated to deliver an irradiance of 15 mW/cm^2^. The 385 nm LED exhibits a peak at 385 nm with a sharp decline beyond 400 nm, while the 457 nm LED peaks at 457 nm, extending slightly past 500 nm. The comparable peak heights and bandwidth indicate comparable balanced power output from both LEDs, suitable for wavelength-specific experiments. Importantly, the distinct spectral regions show that the blue LED does not emit in the UVA range, and the 385 nm LED does not extend into the visible blue spectrum, ensuring clear separation of UV and visible light in experiments. The figure was created using MATLAB (version R2022a).

An LED-based device was designed to irradiate cells inside an operational cell culture flow hood. This water-cooled high-power light module consisted of 16 blue LEDs (ams-OSRAM United States INC., LZ1-00B202, λpeak = 457 nm ± 20 nm bandwidth) and 16 UVA LEDs (ams-OSRAM United States INC., LZ1-00UB0RU4, λpeak = 385 nm ± 10 nm bandwidth), arranged in four rows of four LEDs each. The light beam was generated by rows of LEDs positioned above the cells, ensuring uniform illumination across the top plane of each well in the 24-well plate or T75/T25 cell culture flasks (Figure 1). The illumination module was pre-calibrated by fixing a white blank piece of paper at 12 cm, taking a photo, and analyzing the beam’s homogeneity using ImageJ (NIH, United States, Version 1.53 k) (Mignon and Uzunbajakava, 2020). As shown in Figure 2, both the 385 nm and 457 nm LEDs were calibrated at 12 cm distance, with their spectral irradiance profiles confirming distinct peaks at 385 nm and 457 nm, respectively. Before each light treatment, the light module was calibrated using a UNO power meter (Gentec, Quebec, Canada) connected to a silicon-type photodiode (PH100-Si-HA-OD1-D0, Gentec, Quebec, Canada) and then positioned 12 cm from the cells. Sham conditions were set up nearby, with plates wrapped in aluminum foil to replicate environmental conditions without light exposure. To avoid reflection and scattering of photons from the aluminum-like floor of the cell culture hood, an opaque plastic bag was placed beneath the well-plate or flasks. Exposure time and irradiance settings were managed through a controller interface. Prior to each light exposure, cells were washed once with PBS and irradiated in fresh PBS. The lid was carefully removed from the 24-well plate during treatment to ensure direct light exposure. Every light treatment was conducted within the cell culture flow hood to minimize the risk of contamination. After every treatment, cells were replenished with fresh cell culture media.

### Assessing light-induced cell damage and melanogenesis stimulation in primary keratinocytes

**FIGURE 3:**
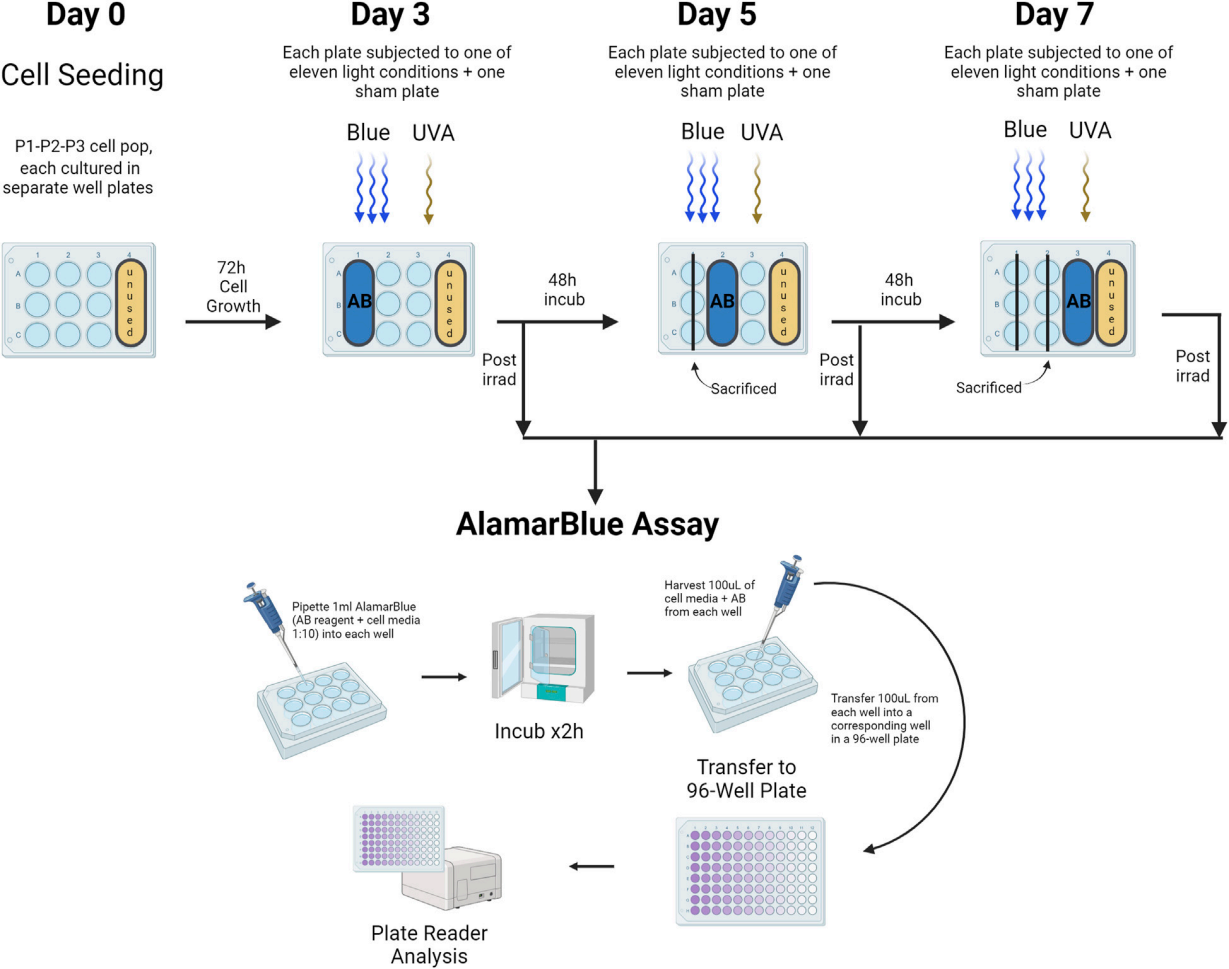
Assessment of Cell Viability Using the AlamarBlue HS Assay. This assay evaluated primary human keratinocyte viability in a 12-well plate format, with distinct plates for both sham and treated groups across different light exposures. Three cell populations (Population 1, Population 2, Population 3) were studied. Viability assessments occurred on days 3, 5, and 7, targeting wells in the first, second, and third rows respectively. Following a 2-h incubation at 37°C, 100 μL from each well treated with AlamarBlue was transferred to a corresponding well in a 96-well plate for analysis via a plate reader. AB = AlamarBlue Assay, P1-P2-P3 = Cell populations 1–3. The figure was created with BioRender.

The AlamarBlue HS assay (Invitrogen) was used as an indirect measure of cell viability and proliferation, as it assesses mitochondrial activity. Keratinocytes were seeded at a density of 6,500 cells/cm^2^ in every well to ensure consistent cell numbers. The assay was mixed with cell media at a ratio of 1:10, and 1 mL of this AlamarBlue solution was added to the cells in a 12-well plate after the designated treatment. Separate plates were used for sham and treated groups, with 9 wells occupied per plate, totaling 22 plates for each cell population (Population 1, Population 2, and Population 3). On day 3, the first row of wells was incubated with AlamarBlue HS, on day 5 the second row, and on day 7 the third row. The remaining wells were replenished with fresh keratinocyte medium. After a 2-hour incubation at 37°C, 100 μL from each AlamarBlue-treated well was transferred to a 96-well plate for measurement using a plate reader (Varioskan, ThermoFisher, Waltham, MA, United States) at 560 nm (excitation) and 590 nm (emission) with 15 flashes and a gain of 85%. Afterward, the cells in the AlamarBlue-treated wells were replenished with PBS and Fungizone and not used further (Figure 3).

**FIGURE 4:**
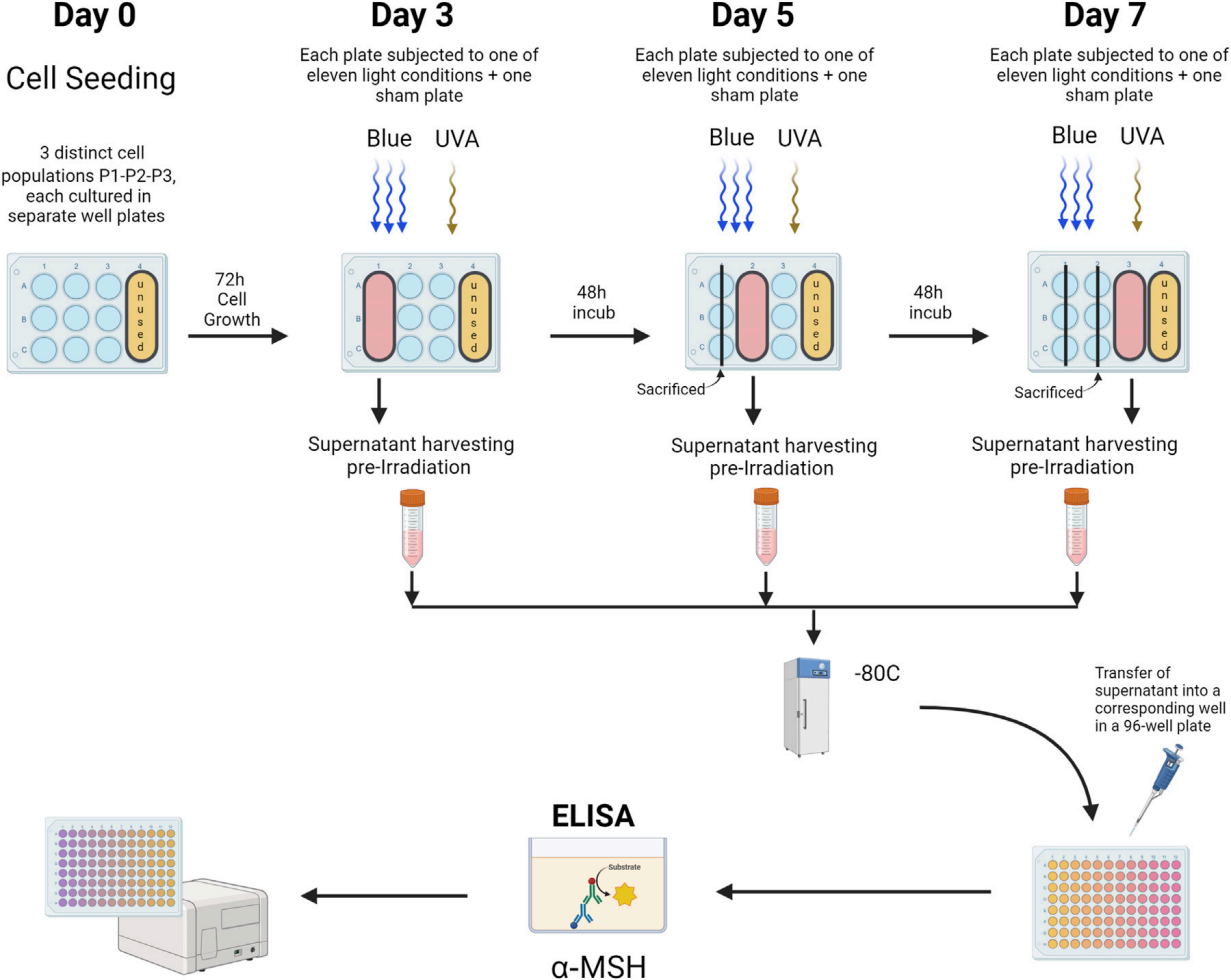
α-MSH ELISA Analysis of Primary Human Keratinocytes. This figure illustrates the ELISA protocol used to evaluate α-MSH levels in cell media from primary human keratinocytes cultured in 12-well plates. Distinct plates were used for sham and treated groups, subjected to various light exposures across 3 cell populations (Population 1, Population 2, Population 3). Prior to each light treatment, media from wells designated for that day’s treatment (as outlined in the plate design) were collected, harvested, and stored at −80°C. Following the completion of treatments for all cell populations, the media samples were thawed on ice and analyzed following the manufacturer’s instructions for the ELISA assay. P1-P2-P3 = Cell populations 1–3. The figure was designed using BioRender.

For the ELISA protocol, α-MSH ELISA testing (LS-F40047 - LS-Bio, MA, United States) was conducted on cell media collected from primary human keratinocytes. Before each treatment and prior to washing the wells with PBS for light exposure, cell media from the wells designated for the treatment day (according to the plate design) were harvested and frozen at −80°C for subsequent analysis. Once all conditions for all three populations (N = 3) were treated, the cell media were thawed on ice and assayed following the manufacturer’s recommendations (Figure 4).

### Sequential Irradiation and keratinocyte-conditioned medium in the culture and analysis of pro-melanogenic factors in melanocytes

Primary keratinocytes were initially cultured separately from melanocytes. On day 0, keratinocytes were seeded at a density of 6,500 cells/cm^2^ onto a feeder layer of mitotically inactivated human fibroblasts, which had been treated with 6,000 rad of gamma radiation. The keratinocytes were irradiated on days 3, 5, and 7 using an LED module with light parameters set to 10 mW/cm^2^ and 15 J/cm^2^. Prior to each irradiation, the culture medium was collected and stored at −80°C for subsequent use in melanocyte culture. The culture medium was collected before each irradiation to allow sufficient time for the keratinocytes to express and release α-MSH, a process that takes several hours. Additionally, the cells were exposed to light in PBS to prevent the absorption of blue light by phenol red and other biomolecules in the culture medium, which could otherwise lead to the generation of reactive oxygen species (ROS) and potentially harm the cells. By collecting the fresh cell medium added after the light treatment, or even only the PBS medium used during irradiation, we would miss the α-MSH that had been released during the day and night prior to light exposure, which is critical for accurately assessing this pro-melanogenic release in keratinocytes.

The collected keratinocyte-conditioned medium was categorized as CM (keratinocyte medium only), CMK (conditioned medium from non-irradiated keratinocytes), or CMiK (conditioned medium from irradiated keratinocytes). After the final irradiation on day 7, the collected keratinocyte-conditioned medium (CM, CMK, or CMiK) was concentrated 10-fold using centrifugal concentrator (Vivaspin 6 centrifugal filtration unit, Sartorius) following the manufacturer’s guidelines, and then stored at −80°C.

**FIGURE 5:**
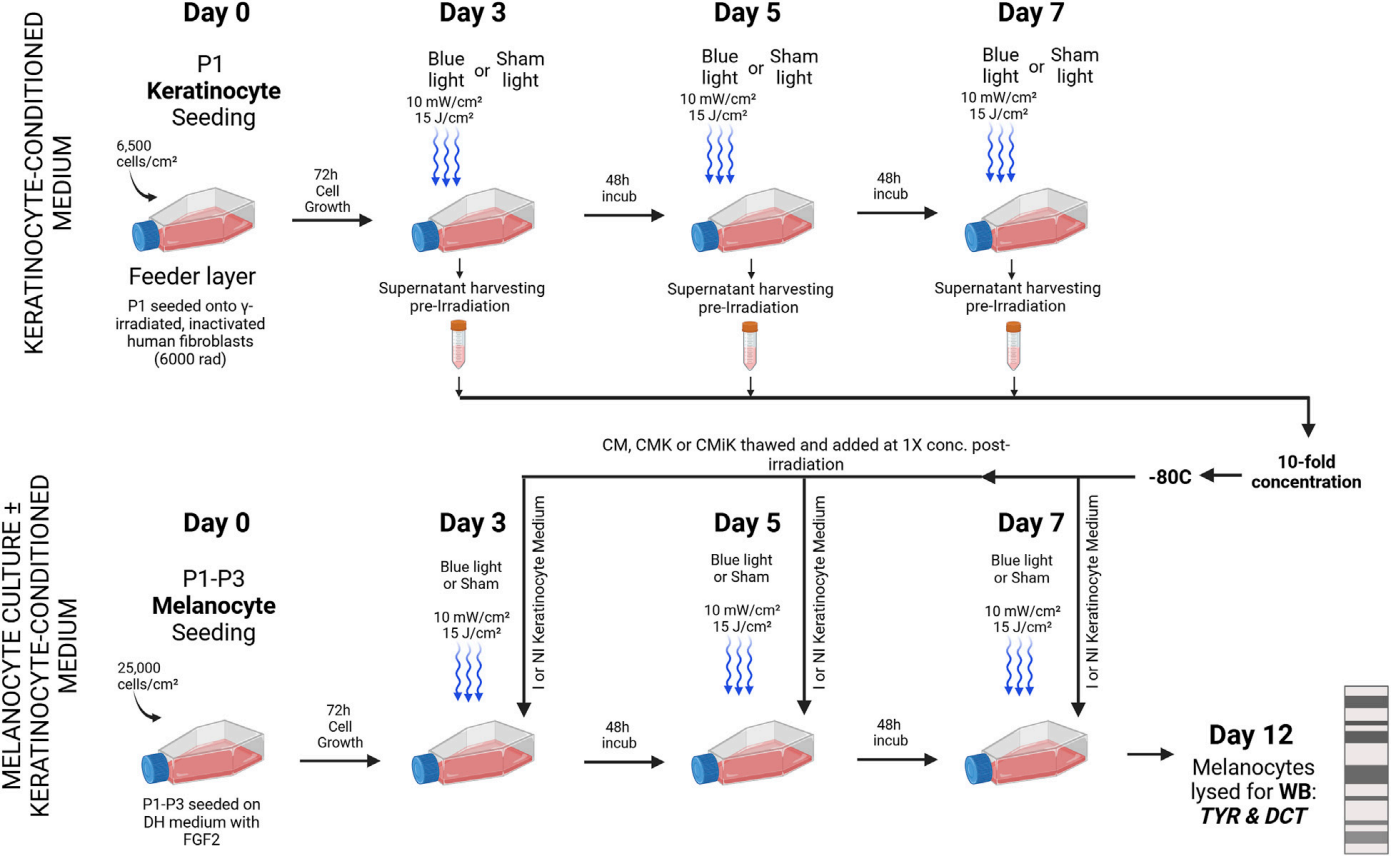
Sequential Irradiation and Medium Exchange in Melanocyte Culture ± Keratinocyte-Conditioned Medium for Pro-Melanogenic Enzyme Analysis. Primary keratinocytes (Population 1) were cultured separately on a feeder layer of gamma-irradiated human fibroblasts and irradiated on days 3, 5, and 7 using an LED module with specified light parameters (10 mW/cm^2^, 15 J/cm^2^). The culture medium was collected prior to irradiation to allow α-MSH accumulation over several hours and because cells were irradiated in PBS to prevent photon absorption and ROS generation. The collected medium was stored at −80°C and later concentrated 10-fold. Melanocytes (Populations 1, 3, and 4), seeded in keratinocyte medium with FGF2, received thawed keratinocyte-conditioned medium at 1X concentration post each irradiation. Three types of conditioned medium were used: CM (keratinocyte medium only), CMK (medium from non-irradiated keratinocytes), and CMiK (medium from irradiated keratinocytes). On day 12, melanocytes were harvested and analyzed via Western blot for TYR and DCT, marking the analysis of pro-melanogenic activity across different light and media conditions. I = Irradiated, NI = non-irradiated, Sham = non-irradiated cell culture, KCM = keratinocyte-conditioned medium, WB = Western blot. This figure was sketched using BioRender.

Melanocytes were seeded on day 0 at a density of 25,000 cells/cm^2^ in keratinocyte medium supplemented with 0.2 nM FGF2. Following each irradiation on days 3, 5, and 7, the keratinocyte-conditioned medium (CM, CMK, or CMiK) was thawed and added at a 1X concentration to the fresh keratinocyte medium supplemented with 0.2 nM FGF2 intended for the melanocytes. On day 12, the melanocytes were harvested and lysed for Western blot analysis to detect the expression of pro-melanogenic factors, including tyrosinase (TYR) and dopachrome tautomerase (DCT) (Figure 5).

### Protein extraction

Melanocyte protein extraction was performed using a lysis buffer containing 1% (v/v) NP40 (Biobasic, Markham, Ontario, Canada), 1% (v/v) Triton-X-100 (Bio-Rad, Hercules, CA, United States), 0.1% (w/v) SDS (Bio-Rad, Hercules, CA, United States), and 0.5% (w/v) Sodium Deoxycholate (Fisher Chemical, Waltham, MA, United States) in PBS. Cells were lysed by adding the lysis buffer, followed by incubation on ice for 10 min. The lysates were then subjected to sonication (450 Digital Sonifier, Branson, Brookfield, CT, United States) at 20% amplitude for 15 s to break cell membranes and shear DNA, repeated for a second cycle, and centrifuged at 13,000 g for 10 min at 4°C. The supernatants were collected, and protein concentrations were determined using a Micro BCA™ Protein Assay Kit (ThermoFisher, Waltham, MA, United States). The lysates were then stored at −80°C until further analysis.

### Western blot analysis

Cell lysates were separated by SDS-PAGE at 80 V for 3 h on polyacrylamide gels (10% acrylamide) under reducing-denaturing conditions (i.e., beta-mercaptoethanol in Laemmli buffer). Following separation, proteins were transferred onto nitrocellulose membranes at 100 V for 1 h at 4°C. The membranes were blocked with Tris-buffered saline containing 0.5% Tween 20 (TBS-T) and 5% (w/v) milk for 30 min. After blocking, the membranes were incubated overnight at 4°C with primary antibodies diluted in blocking buffer: Tyrosinase [T311] (ThermoFisher, 1/100 dilution) and DCT [Ab74-073] (Abcam, 1/1,000 dilution). Following three brief rinses, membranes were washed five times for 10 min each in TBS-T. Secondary antibodies, including goat anti-mouse [62–6520] (Invitrogen, 1/10,000 dilution) and goat anti-rabbit [65–6120] (Invitrogen, 1/10,000 dilution), were applied with agitation for 1 h at room temperature, followed by identical washing steps. Protein detection was achieved using SuperSignal West Dura substrate (ThermoFisher, Waltham, MA, United States) and imaged using the Fusion Fx7 imager (Montreal Biotech Inc., Dorval, QC, Canada). Quantification was performed using ImageJ software (NIH, United States, Version 1.53 k).

### Statistical analysis

All data were first subjected to rigorous testing for normality and sphericity to ensure the appropriateness of subsequent statistical analyses. Normality was assessed using the Shapiro-Wilk test, while sphericity was evaluated with Mauchly’s test. Upon confirmation of these assumptions, repeated-measures ANOVA was conducted, using either a 1-way or 2-way approach depending on the experimental design and variables in question. Post-hoc multiple comparisons were performed with the Tukey correction. A statistical difference was considered significant when the *p*-value was below 5%, ensuring robust and reliable conclusions. All analyses and graphs were performed using R (version 4.4.1), utilizing the packages tidyverse (version 2.0.0), car (version 3.1.2), afex (version 1.3.1), emmeans (version 1.10.3), ggplot2 (version 3.5.1), and dplyr (version 1.1.4).

## Results

### Alamar blue assessment findings

**FIGURE 6:**
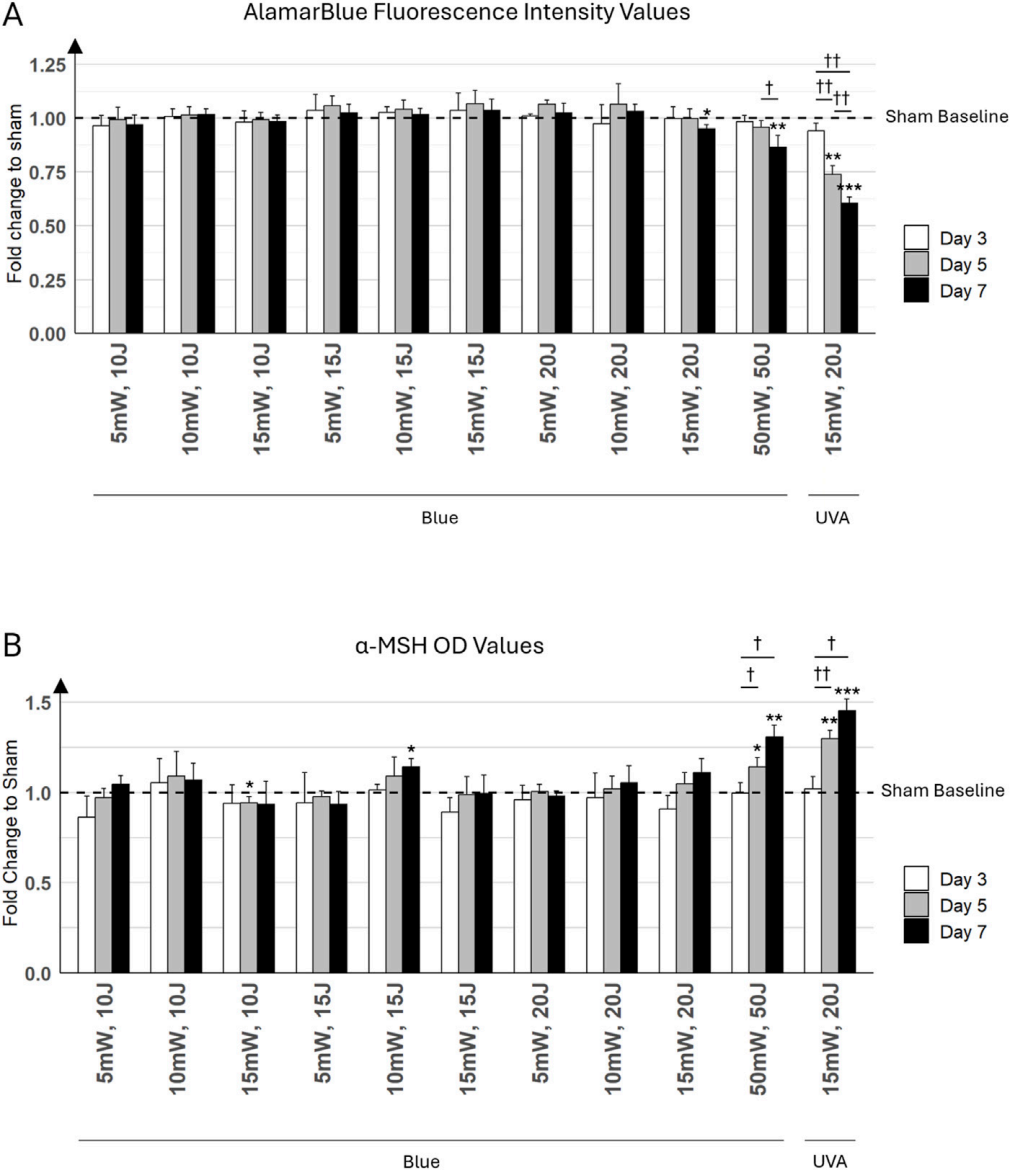
Keratinocyte cultures were exposed to various light parameters across three time points: day 3 (white bars), day 5 (gray bars), and day 7 (black bars). **(A)** Effects of Different Irradiation Parameters on Primary Keratinocyte Viability. This bar graph presents the results of the Alamar Blue (AB) assay conducted on primary keratinocyte monolayers. The *x*-axis displays different combinations of irradiance (mW/cm^2^) and fluence (J/cm^2^) for blue light, with the last combination representing UVA light at 15 mW/cm^2^ and 20 J/cm^2^. The *y*-axis indicates the fold change in cell viability relative to sham control (untreated cells). **(B)** Alpha-MSH Levels in Keratinocyte Cultures Exposed to Various Light Parameters. This graph shows the fold change in alpha-Melanocyte Stimulating Hormone (α-MSH) levels, measured by ELISA. The *x*-axis lists the combinations of irradiance (mW/cm^2^) and fluence (J/cm^2^) for blue light, with the last combination representing UVA light at 15 mW/cm^2^ and 20 J/cm^2^. The *y*-axis displays the fold change in α-MSH levels compared to the sham control (untreated cells). Error bars represent the standard deviation (SD) among the three distinct cell populations (N = 3) analyzed. Statistically significant differences between days for each treatment condition are marked by crosses (^†^ for *p* < 0.05, ^††^ for *p* < 0.01). Statistical differences between a condition (i.e., day and light treatment) and its respective sham are marked by asterisks (* for *p* < 0.05, ** for *p* < 0.01, *** for *p* < 0.001).

A significant reduction in mitochondrial activity was observed in keratinocytes with the strongest light parameter of 50 mW/cm^2^, 50 J/cm^2^, particularly by day 7, where the AlamarBlue signal decreased to 0.86 ± 0.055 (mean ± SD), representing a fold decrease compared to the respective sham (Figure 6A). The decrease in the AlamarBlue signal suggests that cellular metabolism in keratinocytes is diminishing under these conditions. As expected, the UVA light positive control at 15 mW/cm^2^ and 20 J/cm^2^ showed a more pronounced reduction in metabolic activity, dropping to 0.60 ± 0.031 by day 7, also relative to its sham. Minimal impact on metabolism was noticed with lower blue light parameters (5 mW/cm^2^, 10 J/cm^2^ to 10 mW/cm^2^, 20 J/cm^2^), which did not significantly affect the AlamarBlue signal. Interestingly, blue light condition at 15 mW/cm^2^, 20 J/cm^2^ showed a fold decrease of 0.96 ± 0.022, which was very close to the sham. While this difference was statistically significant (*p* < 0.05), it suggests only a minimal reduction in cellular metabolic activity in keratinocytes, indicating almost no cytotoxicity.

### α-MSH induction under various light parameters

Moderate blue light parameters (5 mW/cm^2^, 10 J/cm^2^ to 15 mW/cm^2^, 15 J/cm^2^) did not significantly affect α-MSH levels. In contrast, the highest blue light parameter (i.e., 50 mW/cm^2^, 50 J/cm^2^) resulted in progressive increases in α-MSH levels, particularly noticeable by day 7, reaching 1.31 ± 0.064 (mean ± SD) relative to its sham. Predictably, the UVA condition (15 mW/cm^2^ and 20 J/cm^2^) showed the strongest stimulation of α-MSH production, with a significant and pronounced increase from day 3 to day 7, confirming the potent effect of UVA light on α-MSH induction (Figure 6B). Specifically, on day 7, the UVA condition reached a fold increase of 1.45 ± 0.066 compared to its sham.

On day 7, α-MSH levels under 10 mW/cm^2^, 15 J/cm^2^ reached a fold increase of 1.14 ± 0.046 relative to its sham, while the 15 mW/cm^2^, 20 J/cm^2^ condition resulted in a similar fold increase of 1.11 ± 0.075. However, despite the comparable levels of α-MSH production between these two conditions, the AlamarBlue assay started to show a decrease in metabolic activity under the 15 mW/cm^2^, 20 J/cm^2^ condition compared to the 10 mW/cm^2^, 15 J/cm^2^ condition by day 7.

Given that the highest blue light combinations (15 mW/cm^2^, 20 J/cm^2^ and 50 mW/cm^2^, 50 J/cm^2^) showed a decrease in cell metabolic activity, possibly reflecting cell damage in AlamarBlue assays, the 10 mW/cm^2^, 15 J/cm^2^ combination was selected for further experiments in the co-culture model. This decision was made because it demonstrated a strong potential for inducing high α-MSH release, while also maintaining adequate cell metabolic activity. This allowed for a clearer investigation into α-MSH induction without the confounding effects of cellular stress or damage.

**FIGURE 7:**
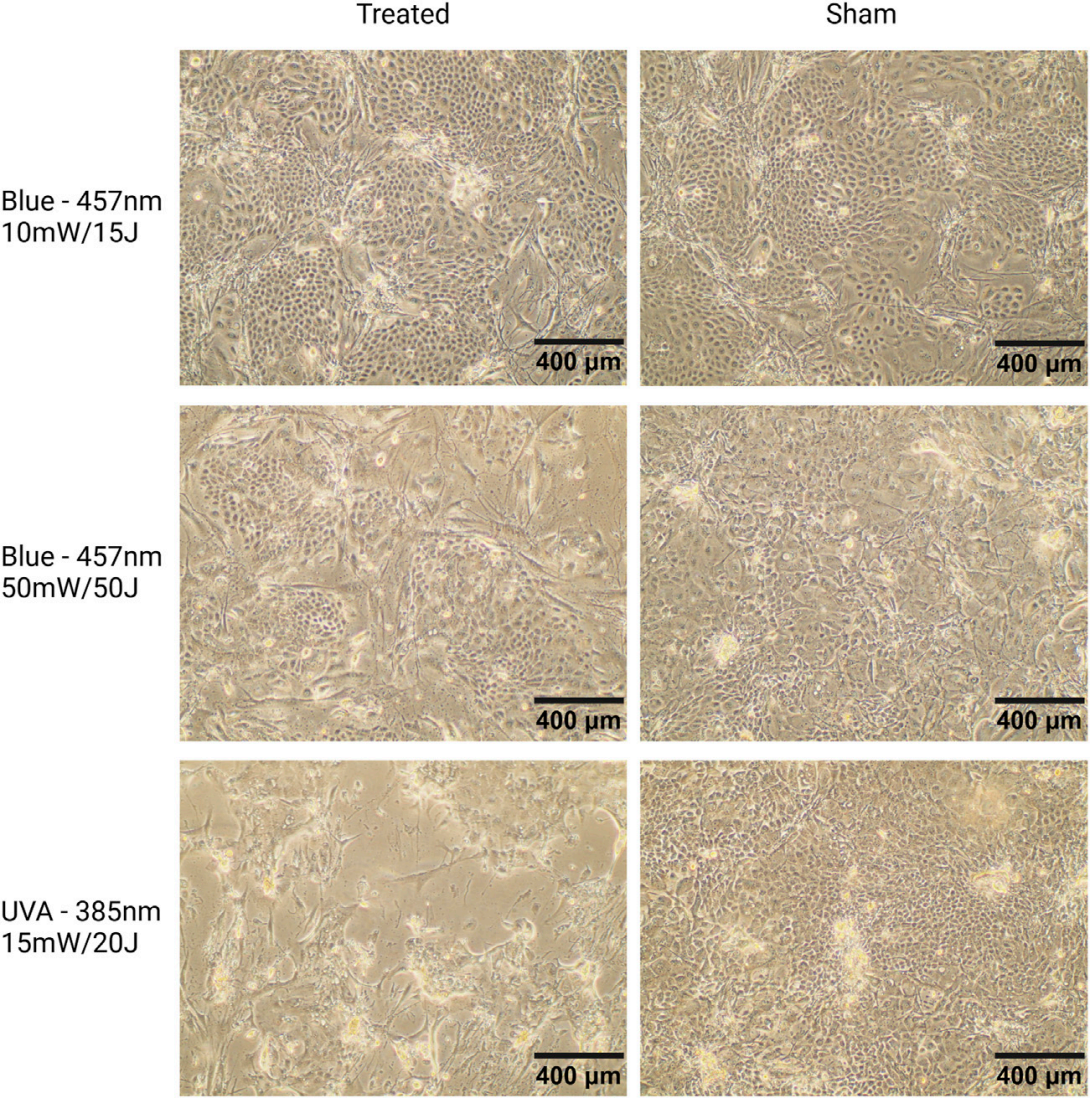
Microscopic images of keratinocyte cultures seeded on a feeder layer of mitotically inactivated fibroblasts, captured at 10X magnification. Representative images from donor K3 on day 7. Cells were exposed to blue light (457 nm, 10 mW/cm^2^, 15 J/cm^2^; 50 mW/cm^2^, 50 J/cm^2^) or UVA light (385 nm, 15 mW/cm^2^, 20 J/cm^2^) on days 1, 3, and 7. Keratinocytes treated with 10 mW/cm^2^, 15 J/cm^2^ (top left) show minimal morphological changes compared to sham, reflecting the low cytotoxicity of these parameters. Keratinocytes exposed to 50 mW/cm^2^, 50 J/cm^2^ blue light (middle left) form smaller but well-organized colonies, consistent with blue light’s known effects of reducing keratinocyte proliferation and promoting differentiation (Castellano-Pellicena et al., 2019). UVA-treated cells (lower left) display signs of damage, including cell shrinkage, nuclear condensation, and membrane disruption. Scale bars = 400 µm.

*Differential Effects of UVA and Blue Light on Keratinocyte Morphology and Cytotoxicity* We observed a distinct difference between the effects of UVA and blue light (Figure 7), which highlighted the boundary between non-ionizing and ionizing radiation. Under the microscope, progressive cell damage from day 3 to day 7 was evident in keratinocyte cultures exposed to UVA, including signs such as cell detachment, morphological changes, and reduced cell density. In contrast, keratinocytes exposed to blue light at the highest parameters (50 mW/cm^2^, 50 J/cm^2^) displayed less pronounced damage, maintaining more organized colonies with only moderate structural changes. Notably, keratinocytes treated with lower blue light parameters (10 mW/cm^2^, 15 J/cm^2^) exhibited minimal morphological alterations compared to sham controls, with cells forming well-organized colonies and showing no significant signs of structural deterioration. These findings are consistent with the observed low cytotoxicity of lower-intensity blue light in the AlamarBlue analysis (Figure 6A).

### Western blot analysis of tyrosinase and DCT

#### Tyrosinase

**FIGURE 8:**
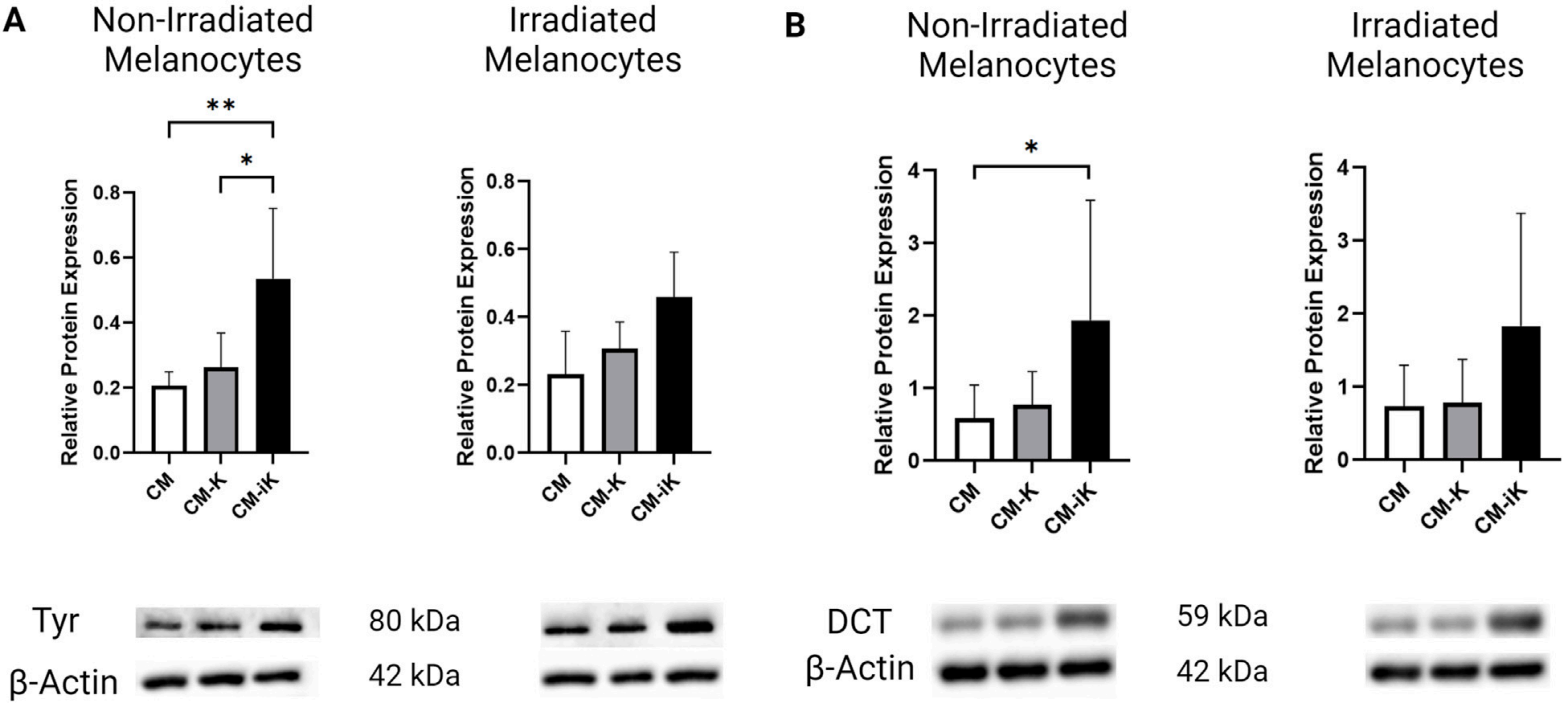
Relative protein expression of Tyrosinase (Tyr) and Dopachrome Tautomerase (DCT) normalized to β-actin in non-irradiated and irradiated primary melanocytes cultured with or without keratinocyte-conditioned medium. **(A)** Tyrosinase Protein Expression: Bar graphs show fold change in Tyr levels in non-irradiated (left) and irradiated (right) melanocytes. Conditions include melanocytes without keratinocyte-conditioned medium (CM), with conditioned medium from non-irradiated keratinocytes (CM-K), and irradiated keratinocytes (CM-iK). Significant increases in Tyr expression were observed in non-irradiated melanocytes exposed to irradiated keratinocyte-conditioned medium (CM-iK) (*p* < 0.05). Immunoblot images of Tyr and β-actin are shown below the bar graphs. **(B)** Dopachrome Tautomerase (DCT) Protein Expression: Bar graphs present the fold change in DCT levels in non-irradiated (left) and irradiated (right) melanocytes. A significant increase in DCT expression is seen in non-irradiated melanocytes exposed to irradiated keratinocyte-conditioned medium (*p* < 0.05). Immunoblot images of DCT and β-actin are shown below the bar graphs. Error bars indicate the standard deviation (SD) between the three populations of melanocytes (N = 3) assessed, and *p* < 0.05 is considered statistically significant.

Blue light irradiation at 10 mW/cm^2^, 15 J/cm^2^ significantly increased Tyrosinase levels in melanocytes. Additionally, in non-irradiated melanocytes, the addition of conditioned medium from irradiated keratinocytes (CM-iK), which were cultured for 7 days and exposed to light 3 times, further enhanced Tyrosinase expression during the 12-day culture period. This suggests that factors released by irradiated keratinocytes contribute to this increase in Tyrosinase expression (Figure 8A). Moreover, while irradiated melanocytes also displayed elevated Tyrosinase levels, the increase was less pronounced compared to non-irradiated melanocytes treated with CM-iK.

#### DCT

The conditioned medium from non-irradiated keratinocytes (CM-K) slightly increased DCT expression in melanocytes, while the irradiated keratinocyte-conditioned medium (CM-iK) further stimulated DCT expression. Regardless of whether the melanocytes were irradiated or not, DCT expression remained low in the absence of keratinocyte-conditioned medium. However, the addition of conditioned medium—especially from irradiated keratinocytes—led to a significant increase in DCT levels, with CM-iK conditions showing the highest DCT expression for both non-irradiated and irradiated melanocytes (Figure 8B). Notably, the increase in DCT expression was statistically significant (*p* < 0.05) in non-irradiated melanocytes treated with CM-iK compared to the condition without keratinocyte-conditioned medium (CM).

#### Microscopic observations of melanocytes cultured in CM, CMK, and CMiK

**FIGURE 9:**
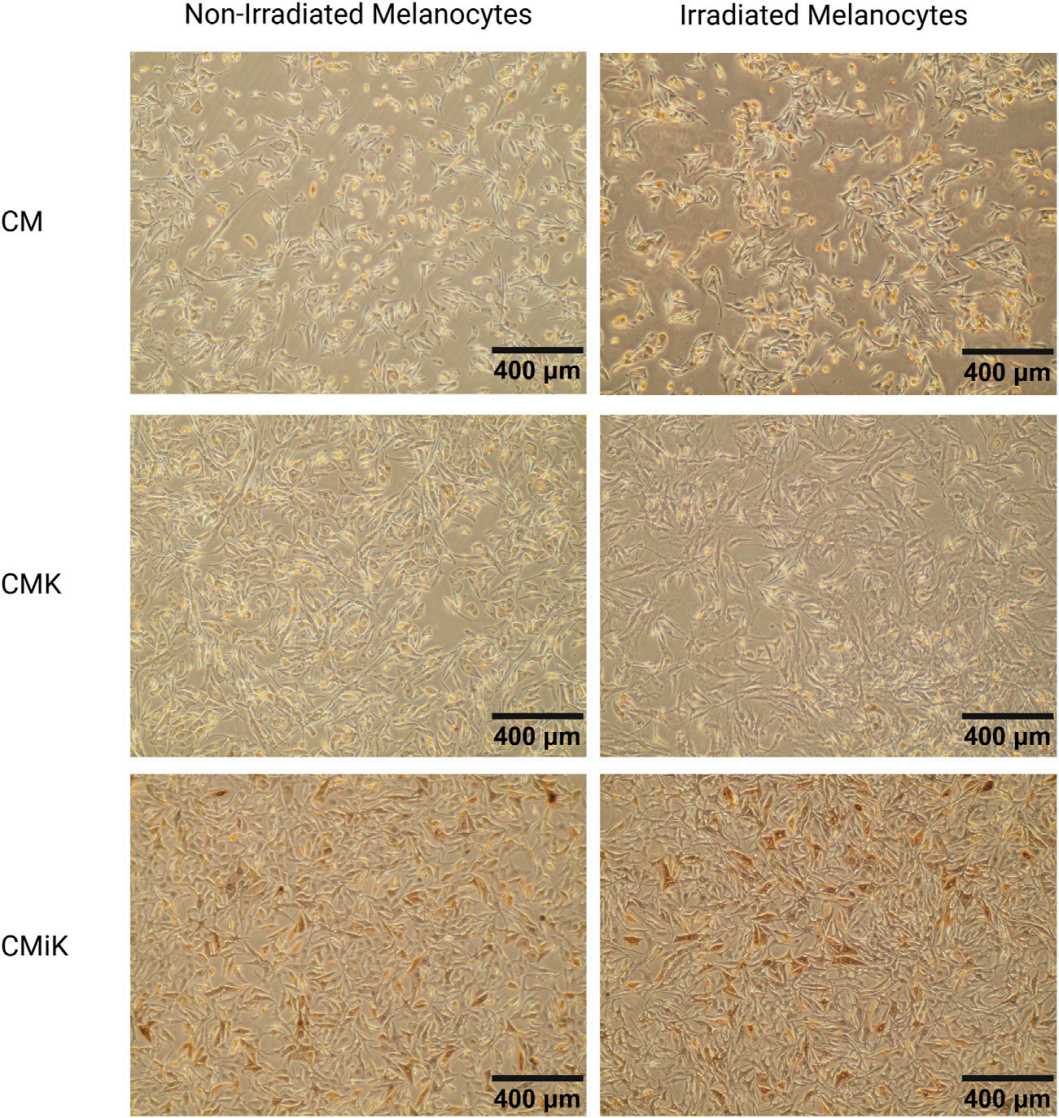
Microscopic images of melanocytes (population 1) cultured in conditioned media from keratinocytes (CM, CMK, CMiK) before lysis for Western blot analysis on day 12. Representative images of melanocytes cultured in CM (keratinocyte medium only), CMK (conditioned medium from non-irradiated keratinocytes), and CMiK (conditioned medium from irradiated keratinocytes) are shown for non-irradiated (left panels) and irradiated (right panels) melanocytes. Images were captured at ×10 magnification. Melanocytes in CM show a healthy morphology with well-defined spindle shapes and minimal pigmentation. Those cultured in CMK exhibit slightly more spindle elongation with no noticeable pigmentation changes. Melanocytes cultured in CMiK display enhanced pigmentation, particularly in irradiated conditions, consistent with increased pro-melanogenic factor activity. These photomicrographs were obtained from live-cell cultures without fixation, eliminating the possibility of formalin or other pigment artifacts. Scale bars = 400 µm.

Representative images of melanocytes cultured in CM, CMK, and CMiK before lysis for WB analysis on day 12 reveal condition-dependent differences in morphology, pigmentation, and apparent proliferation (Figure 9). Melanocytes cultured in CM exhibited healthy spindle-shaped morphology with no noticeable pigmentation differences between irradiated and non-irradiated conditions. In contrast, those cultured in CMK showed slightly more elongated spindle morphology with a visually apparent increase in proliferation, though not quantified, and no significant pigmentation changes. Notably, melanocytes cultured in CMiK displayed a pronounced increase in pigmentation, particularly under irradiated conditions, along with a visible increase in cell density, suggesting enhanced proliferation. These observations are consistent with the pro-melanogenic activity of irradiated keratinocyte-conditioned medium, aligning with the biochemical findings from WB analysis (Figure 8). These results visually reinforce the differential effects of CM, CMK, and CMiK on melanocyte morphology, pigmentation, and proliferation, highlighting the contribution of irradiated keratinocytes to melanogenesis and cell growth.

## Discussion

Our study demonstrates how different blue light parameters (5 mW/cm^2^ to 50 mW/cm^2^) affect both cellular metabolic activity and melanogenic responses in primary cultured human keratinocytes and melanocytes. The key findings in this study indicate that (i) moderate blue light (10 mW/cm^2^, 15 J/cm^2^) maintains cell metabolic activity while inducing a modest increase in α-MSH production, (ii) the highest blue light parameter (50 mW/cm^2^, 50 J/cm^2^) leads to a significant reduction in metabolic activity, suggesting cytotoxicity, and (iii) keratinocyte-conditioned medium from irradiated cells promotes enhanced melanogenesis in melanocytes, highlighting the paracrine signaling role of keratinocytes in this pathway. While only specific moderate blue light conditions (10 mW/cm^2^, 15 J/cm^2^) induced a significant increase in α-MSH production, other parameters showed minimal effects on α-MSH levels. These results underscore the importance of selecting appropriate light parameters to maximize therapeutic benefits while minimizing adverse effects.

### Balancing blue light intensity for optimal cell viability and melanogenesis: a dose-response study on keratinocytes and melanocytes

The Alamar Blue assay showed that most blue light parameters did not significantly reduce cellular metabolic activity, except for the highest intensity (50 mW/cm^2^, 50 J/cm^2^), as well as the UVA condition (15 mW/cm^2^, 20 J/cm^2^). The severe reduction in metabolic activity under UVA light, which served as a positive control, emphasizes the known cytotoxic effects of UVA due to reactive oxygen species (ROS) generation (Matsumura and Ananthaswamy, 2004). The decrease in cellular metabolism at higher blue light intensities suggests increased cytotoxicity, often indicative of cell stress or damage (Gonzalez and Tarloff, 2001; O'Brien et al., 2000). In contrast, moderate blue light intensities (5 mW/cm^2^, 10 J/cm^2^ to 10 mW/cm^2^, 15 J/cm^2^) preserved cellular metabolism, confirming these parameters fall within a safe exposure threshold.

The α-MSH ELISA results demonstrated that light parameters had a distinct influence on α-MSH production in keratinocytes. Lower blue light intensities maintained α-MSH levels similar to the control, indicating minimal melanogenic stimulation. As intensity and fluence increased, α-MSH levels were enhanced, particularly by day 7. The UVA condition triggered the most robust α-MSH response, underscoring its strong effect on melanogenesis, likely via the canonical melanogenesis pathway (Naikoo et al., 2023). However, the reduction in metabolic activity at the highest blue light intensities points to a trade-off between effective α-MSH induction and cytotoxicity. This prompted the selection of the intermediate blue light condition (10 mW/cm^2^, 15 J/cm^2^) for further experiments, balancing therapeutic efficacy and cellular safety.

The dose-dependent nature of cellular responses to light exposure was evident from the biphasic pattern observed in both the Alamar Blue and α-MSH results. While lower intensities preserved metabolic activity and α-MSH production at baseline levels, intermediate intensity (10 mW/cm^2^, 15 J/cm^2^) optimally stimulated α-MSH production with minimal cytotoxic effects. In contrast, the highest intensity (50 mW/cm^2^, 50 J/cm^2^) led to significant reductions in metabolic activity, highlighting the importance of carefully calibrating light parameters to harness the therapeutic potential of photobiomodulation (PBM) without causing harm.

Photobiomodulation (PBM) is a complex process that requires careful calibration of parameters such as wavelength, irradiance, and fluence to achieve optimal biological effects (Barolet, 2021). In our study, significant reductions in metabolic activity were only observed at the highest blue light intensity (50 mW/cm^2^, 50 J/cm^2^) and under UVA exposure. However, a slight decrease was also noted at 15 mW/cm^2^, 20 J/cm^2^ by day 7, suggesting potential cellular stress at this level. The gap between these two intensities leaves the exact limits of a “safe window” undetermined. Although 10 mW/cm^2^, 15 J/cm^2^ was identified as an optimal balance, showing enhanced α-MSH production while maintaining cell metabolic activity, further investigation is needed to more precisely define the safety thresholds. These findings demonstrate that moderate blue light parameters can be effectively leveraged for safe and effective treatments, though continued refinement is essential.

### Modulation of melanogenic proteins by blue light irradiation and keratinocyte medium

Our results underscore the significant role of blue light irradiation in modulating the expression of key melanogenic proteins, such as Tyrosinase and DCT in melanocytes. The observed increase in these protein levels following irradiation suggests direct activation of melanogenic pathways. Furthermore, the conditioned medium of keratinocytes, particularly after their irradiation, significantly enhanced the expression of Tyrosinase and DCT, highlighting the release of stimulatory factors that increase melanocyte responses.

These findings demonstrate that tyrosinase and DCT are reliable markers of melanogenesis, as their expression directly correlates with melanin synthesis in melanocytes. This is further validated by photomicrography (Figure 9), which visually confirms increased pigmentation corresponding to elevated tyrosinase and DCT levels (Figure 8). Importantly, the photomicrographs were obtained without fixation techniques, such as formalin, thereby eliminating the possibility of pigment artifacts and ensuring that the observed pigmentation is attributable to melanin production. This methodology aligns with established literature where tyrosinase expression and visual assessments are widely recognized as robust indicators of melanogenesis (Regazzetti et al., 2018; Shin et al., 2012; Hearing and Tsukamoto, 1991).

Although melatonin has been reported to influence melanogenesis under specific conditions, such as in an *ex vivo* eyelid skin model (Sevilla et al., 2023), these findings are preliminary and limited to supraphysiological concentrations, which differ fundamentally from the robust and well-documented role of α-MSH in UV-induced and physiological melanogenesis through tyrosinase activation.

In addition, many studies in the field rely on single-donor primary cells or immortalized cell lines, which can limit the generalizability of their findings (Regazzetti et al., 2018; Costin and Hearing, 2007). By contrast, our study incorporated primary cells from four diverse donors, representing a range of Fitzpatrick phototypes and anatomical sites. This diversity ensures a broader representation of human skin biology and exceeds the standard for comparable studies. As a result, despite the inherent limitations of *in vitro* research, our findings provide a robust foundation for understanding melanogenic responses to blue light.

### Minimized influence of feeder fibroblasts on α-MSH production and melanogenesis: a limitation

Fibroblasts are known to secrete factors that can influence melanogenesis, but their contribution to α-MSH production is negligible compared to keratinocytes (Russo et al., 2020). In this study, mitotically inactivated fibroblasts were used as a feeder layer at a consistent density of 8,000 cells/cm^2^ (approximately 600,000 cells per T75 flask) across all conditions cultured with conditioned keratinocyte media, ensuring uniform baseline contributions. Keratinocytes, as the primary producers of α-MSH, outnumbered fibroblasts by a ratio of approximately 12–13, reaching 7 to 8 million cells per T75 flask after 7 days of culture. Additionally, fibroblasts inactivated by gamma radiation are progressively replaced by the rapid proliferation of keratinocytes, with 60%–70% of the feeder layer no longer present after 7 days of culture (Le-Bel et al., 2019; Rochon et al., 2009). This diminishing presence, combined with the inherently lower α-MSH expression levels of fibroblasts, further reduces their potential impact on the results. While the experimental setup was designed to minimize fibroblast influence, the absence of direct controls to assess fibroblast-specific contributions remains a limitation of this study. Future investigations should explore the role of fibroblast-secreted factors in co-culture systems to better understand their potential effects on melanogenesis under irradiated and non-irradiated conditions.

### Limitations in melanin type differentiation

The primary objective of this study was to establish the cell damage threshold of non-ionizing blue light on keratinocytes while preserving their ability to stimulate melanogenesis. While we observed robust evidence of melanogenesis, the study did not differentiate between eumelanin and pheomelanin. Future studies could explore this distinction using analytical techniques like high-performance liquid chromatography (HPLC), but such analyses are beyond the scope of the present work.

### Translational considerations

When interpreting these findings, it is essential to account for the differences in light propagation between *in vitro* and *in vivo* environments. *In vitro* conditions feature minimal light attenuation in a translucent medium, while *in vivo* settings involve significant photon attenuation due to absorption and scattering in the turbid medium of skin, particularly at 450 nm (Mignon et al., 2018). This highlights the need for accurate translation of irradiance levels to ensure clinical relevance. Clinically, the minimal pigmentation doses for blue light can be remarkably low, with values as low as 10 J/cm^2^ for blue light and 18 J/cm^2^ for green light inducing detectable pigmentation in certain skin types within 30 min (Mahmoud et al., 2010; Falcone et al., 2018). These considerations are critical when designing light-based therapeutic protocols to balance efficacy with safety.

## Conclusion

In conclusion, our study shows that blue light irradiation significantly influences mitochondrial metabolic activity and α-MSH secretion in keratinocytes, as well as melanogenic protein expression in melanocytes. The AlamarBlue assay revealed that cytotoxicity was only evident at the highest blue light intensity (50 mW/cm^2^, 50 J/cm^2^) and UVA exposure, with UVA having the most pronounced effect. However, a slight decrease in metabolic activity was observed at 15 mW/cm^2^, 20 J/cm^2^ on day 7, indicating potential early signs of cellular stress at this level. Moderate blue light intensities (5 mW/cm^2^, 10 Jcm^2^ to 10 mW/cm^2^, 15 J/cm^2^) were well-tolerated, maintaining metabolic activity without significant cytotoxicity.

The α-MSH results highlighted a dose-dependent increase in production, with the highest levels observed under UVA exposure, but moderate blue light also stimulated melanogenesis. These findings suggest that the “safe and effective” window for blue light treatments may lie below 50 mW/cm^2^, but further investigation is needed to refine this range, particularly between 15 mW/cm^2^, 20 J/cm^2^ and 50 mW/cm^2^, 50 J/cm^2^.

Additionally, the regulation of key melanogenic proteins (Tyrosinase and DCT) by blue light and the role of keratinocyte-conditioned medium in promoting melanogenesis underscores the complex interaction between keratinocytes and melanocytes. This study provides a foundation for developing light-based therapies aimed at pigmentation disorders, emphasizing the need for precise parameter optimization to maximize therapeutic outcomes while minimizing potential adverse effects.

## Data Availability

The raw data supporting the conclusions of this article will be made available by the authors, without undue reservation.
